# Making the future together: Shaping autism research through meaningful participation

**DOI:** 10.1177/1362361318786721

**Published:** 2018-08-10

**Authors:** Sue Fletcher-Watson, Jon Adams, Kabie Brook, Tony Charman, Laura Crane, James Cusack, Susan Leekam, Damian Milton, Jeremy R Parr, Elizabeth Pellicano

**Affiliations:** 1 The University of Edinburgh, UK; 2 Flow Observatorium, UK; 3 Autism Rights Group Highland, UK; 4 King’s College London, UK; 5 University College London, UK; 6 Autistica, UK; 7 Cardiff University, UK; 8 Participatory Autism Research Collective, UK; 9 University of Kent, UK; 10 Newcastle University, UK; 11 Macquarie University, Australia

**Keywords:** autism, community, disability rights, involvement, methods, participatory research

## Abstract

Participatory research methods connect researchers with relevant communities to achieve shared goals. These methods can deliver results that are relevant to people’s lives and thus likely to have a positive impact. In the context of a large and growing body of autism research, with continued poor implementation, and some evidence of community dissatisfaction, there is a powerful case for participatory autism research. In order to develop a framework for such collaborative working, a UK seminar series was organised and co-produced by autistic and non-autistic people with academic, practitioner and lived expertise. This article reports on the outcomes from the series, identifying five topics relevant to building a community of practice in participatory research: Respect, Authenticity, Assumptions, Infrastructure and Empathy. Each topic is connected to a specific example from within and beyond research, to inspire new practices in the field. We call for the development of participatory research skills among the autism research community and the facilitation of greater autistic leadership of, and partnership in, research. Such work, if delivered to a high standard, is likely to lead to better translation into practice and improved outcomes for autistic people and those who support them.

## Introduction

Autism research has seen tremendous growth over the last decade (Interagency Autism Coordinating Committee (IACC), 2013; Pellicano et al., 2013). This research has the potential to transform the lives of autistic people^1^ and their families, when it is relevant, valued and effectively implemented. Yet, efforts to apply research findings in public services and professional support have not always been forthcoming, raising serious questions about the utility of past and existing models of practice in autism research (Milton and Bracher, 2013; Pellicano et al., 2014b; Pellicano and Stears, 2011). Participatory research enables meaningful input from autistic people in autism research. It is one important way to overcome barriers to effective translation and to ensure that research yields relevant benefits (Long et al., 2017).

By *participatory research*, we mean incorporating the views of autistic people and their allies about what research gets done, how it is done and how it is implemented (Cornwall and Jewkes, 1995). A key principle of participatory research is the recognition, and undermining, of the traditional power imbalance between researcher and participant (Nelson and Wright, 1995). One way to conceptualise this power imbalance is using Arnstein’s *ladder of participation* – a visual metaphor which explicitly illustrates different types of participation in terms of increasing power (Arnstein, 1969). These range from no power (e.g. recipient of therapy), through tokenism (e.g. informing and consultation), to devolved power (e.g. partnership and citizen control). This influential model has been critiqued, among others, for its failure to recognise that participation itself can be a goal and that *process* and *diversity of experience* matter as much as *outcome* (Tritter and McCallum, 2006). While these comments have clear relevance to autism, especially when considering ways to include autistic people with learning disabilities, the ladder remains a useful shorthand. We would currently conceptualise much of autism research as involving no power, or only tokenistic forms of power, for the autistic community and their allies (Nicolaidis et al., 2011).

Specific manifestations of participatory research might include *leadership* by autistic researchers, *partnership* with autistic people or allies as co-creators of knowledge, *engagement* with the community in general (e.g. via social media) and *consultation* with relevant individuals or community organisations. Another key feature of participatory research is *inclusiveness* including adapting the research environment, methodology and dissemination routes to permit the widest and most accessible engagement, or engagement from specific groups (e.g. non-speaking autistic people and people with additional intellectual disabilities – see Long and Clarkson, 2017). Participatory research is ethically informed by the values of the community, for example, in the selection of research questions and study objectives. Moreover, input from this community can improve the quality of research methods, contextualise findings within real-world settings and thereby enhance the translation of findings into practice (Carrington et al., 2016; Grinker et al., 2012; Parr, 2016; Parsons and Cobb, 2013). However, there is evidence that this engagement is not yet prevalent in the field.

The UK report *A Future Made Together* (Pellicano et al., 2013) sought the views of autistic people and their families, researchers and practitioners (including people identifying with multiple such categories) about their experiences of being involved in research. One key finding from the report was that research funding and output in the United Kingdom does not align with the views of autistic people, family members and practitioners on what research questions should be prioritised – a clear barrier to translation. Views on the prevalence of participatory research were contrasting – while researchers perceived themselves to be engaged with the autism community in both dissemination and discussions about their research, community members, most notably autistic people and their families, did not share this view (Pellicano et al., 2014b).

Successful participatory research requires both cultural and structural changes (Raymaker and Nicolaidis, 2013). Cultural issues include the fact that non-autistic researchers and funders in the field have traditionally seen the primary role of autistic people as participants in research studies (the ‘subjects’ of research). Involving autistic people in active and powerful research roles may be seen to compromise the scientific integrity of the project. Structural issues include the combined effect of general barriers to autistic employment (Lorenz et al., 2016) together with the competitive funding and job market of academia. For example, skilled mentoring and support, essential to post-graduate study and career development for autistic researchers, may be in short supply (Ridout, 2018; Ridout and Edmondson, 2017). For autistic people and family members who are not researchers, there are few opportunities to have meaningful input into decisions about what research gets funded. Put bluntly, the traditional autism research culture – in common with many fields of scientific enquiry (Nicolaidis and Raymaker, 2015) – is inadequate regarding the extent to which autistic people have been able to shape the research agenda, its implementation and dissemination of its findings.

Fortunately, there has been increasing recognition internationally that this situation needs to change, with autistic advocates, academics and activists being some of the strongest voices to speak to these issues (Michael, 2016; Milton, 2014; Nicolaidis et al., 2011; Pellicano et al., 2011). There are signs of a much-needed improvement, from openly discussing these issues (Wright et al., 2014), to communities of researchers and autistic people beginning to enact change (Stahmer et al., 2017). In this article, we report on a seminar series, jointly developed and hosted by people from the autistic and research communities, which aimed to move the field forward by identifying barriers to, and solutions for, participatory autism research. The series itself also provided an opportunity to develop models of good practice in co-creation of knowledge.

### The shaping autism research seminar series

We received funding from the UK’s Economic and Social Research Council (ESRC) to hold a series of seminars to discuss these very issues and determine how autistic people and their allies could shape the future of autism research and practice (Table 1). Seminars were organised, hosted, attended and led by a wide and diverse group. This included researchers (autistic and non-autistic), stakeholders from the *autistic community* (i.e. autistic people including those with an autism spectrum diagnosis and those who self-identify) and their allies – the broader *autism community* – including family members, education and healthcare professionals; third-sector organisations, commissioners and policy-makers; and autism research funders. Many people fell into multiple categories: for example, autistic parents of autistic children; autistic education, healthcare or social care practitioners (Table 1).

**Table 1.** The seminars.

	Topic	Title	Location
1	Autism practice	Developing and sharing approaches to research informed practice for children and young people	Edinburgh
2	Public services	Developing more effective health and social care services in partnership with the autism community	Newcastle
3	Public services	Developing more effective public services in partnership with the autism community	Cardiff
4	Autism and society	Doing autism research well – building a participatory framework for autism research	London
5	Autism and society	Autistic well-being	London
6	Autism practice	Learning and sharing lessons on how to conduct autism research well	London

During the series, our overarching goal was to examine how autism research could become more *participatory* in nature (Israel et al., 2005). One result of participatory research should be that research activities and findings are more *meaningful* – that is, relevant to the community, consistent with their values, and not tokenistic in delivery. Thus, the seminars strove to identify, highlight and embody models of best practice, sharing examples of real and meaningful participatory research from which others could learn. We also sought to identify both barriers to, and possibilities of, more inclusive models of working between autistic people and researchers.

Across 3 years, we held six seminars on three overlapping research areas: *Autism Practice, Public Services* and *Autism and Society* (Table 1). These areas had been identified in *A Future Made Together* (Pellicano et al., 2013) as needing further attention from the research community, relative to more basic science areas and, critically, were also highlighted as priorities for the autism community. At each seminar, we included autistic people (researchers and community leaders) in the planning, organisation and delivery of the seminars, and featured a mixture of local, national and international speakers. We also had a special emphasis on the next generation of autistic and non-autistic early career researchers with the aim of capacity building and improving the future research landscape in the United Kingdom. In total, approximately 200 people were involved in some way in the series with further reach orchestrated via social media (#shapeARUK). Across the seminars, there was remarkable consistency of opinion among delegates about the need for, barriers to, and best practice models for, participatory autism research. The resulting key considerations in effective participatory working for autism research are presented here, grouped under five topic headings.

### Key topics in participatory autism research

The final seminar in the series was a 1-day meeting to discuss methods and forms of participatory working. While the first five seminars were large open events, the final seminar meeting was for a small group of seminar leaders, and selected community representatives and academics. It was attended by 12 people, including 5 who were autistic, 3 who were parents of autistic children, 3 who were working practitioners from clinical and community services and 10 who were academics – with substantial overlap between categories in all combinations. The five topics described below emerged from an iterative discussion process at the meeting, supported by an additional three facilitators. A sub-set of the original group, including autistic and non-autistic people from within and outside academia, are now co-authors on this article.

**Figure 1.**
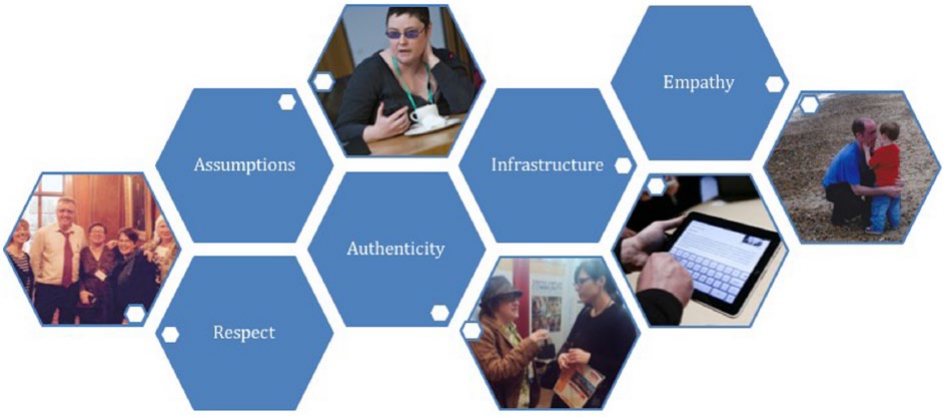
Current topics in participatory autism research.

The discussion concentrated on complex issues in participatory research, aiming to challenge the thinking even of those who are already supportive of the participatory research agenda. Thus, topics selected for elaboration here (see Figure 1) aim to move the debate forward, rather than repeating those (noted elsewhere) which motivated the series (e.g. need for adapted sensory environments; avoidance of deficit models and terminology – see Nicolaidis et al. (2011) and Pellicano and Stears (2011), for an expansion of these topics). Nevertheless, we recognise that some researchers new to participatory working may wish to read more around the background debates that motivated us to propose the seminar series. With such individuals in mind, many relevant resources have been developed and collected at the series website: www.shapingautismresearch.co.uk.

Each topic section includes a single case study (Boxes 1 to 5) highlighting an example from research or practice relevant to the point under discussion. While the first five seminars themselves were organised around domains of working relevant to research, such as practice and public services, the headings emerging from this meeting were based on discussion of *ways* of working which transcend academic disciplines or research targets. Our goal is also to share potential solutions to enable collaborative working, not just to identify barriers. Thus, our chosen exemplars describe methods of participatory working (e.g. autistic leadership and supportive infrastructures) but not necessarily research activities.

**Box 1.** Respect – how to respectfully represent lived experience in research (www.knowyournormal.co.uk).

In the *Know Your Normal*project, a team of autistic volunteers from the UK charity Ambitious about Autism approached academics from the Centre for Research in Autism and Education, University College London, to co-produce research on a topic that they identified as a priority issue – mental health in young autistic people. The team worked in partnership to design the study, conduct the research, and analyse and interpret the data, and write-up and disseminate the results; with the academic researchers ensuring that the research was methodologically and ethically sound, and the autistic volunteers ensuring that the research was relevant and meaningful to the autistic community, representing their lived experience (Crane et al., 2018).	
Strengths of this approach	Limitations of this approach
● Focus on a priority area for autistic people and involvement of autistic co-researchers facilitated recruitment into the study and engagement with findings ● Autistic co-researchers obtained hands-on experience of conducting a research project to completion	● Autistic researchers limited from data collection due to time constraints and personal relationships with participants ● Relied on an approach from an autistic group to get the project off the ground – project would not have happened without their confidence and resourcefulness

**Box 2.** Authenticity – how autism communities can shape a research agenda (www.autistica.co.uk/our-research/your-research-priorities).

Autistica and a consortium of partners launched a James Lind Alliance Priority Setting Partnership to define the top 10 autism research questions. This was an independently facilitated and validated process, which surveyed a representative sample of autistic people, caregivers and professionals before bringing them together to reach agreement on the top 10 through a final workshop. Importantly, the process deliberately excluded researchers from the final workshop so that the top 10 is a genuinely community-led, authentic list (Cusack and Sterry, 2016).	
Strengths of this approach	Limitations of this approach
● Independent facilitation achieved consensus across groups and ensured power balance between groups ● Impact includes increased likelihood of major autism research funding from government and charitable organisations	● Merging perspectives of a diverse group into homogeneous outcomes can result in under-specified priority research topics ● It remains challenging to fully include everyone on the spectrum (e.g. those with additional intellectual disabilities and limited spoken communication)

**Box 3.** Assumptions – best practice in autistic leadership and community advocacy (www.arghighland.co.uk).

Autism Rights Group Highland (ARGH) is a collective of Autistic Adults based in Scotland. They work together to lobby, campaign and educate. Recent activities include a successful campaign to remove puzzle piece imagery from the journal *Autism*. They are now focused on securing continued funding for a local service – the Highland One Stop Shop – providing support to local autistic people and their families encompassing diagnosis and post-diagnostic support, social activities and clubs, and guidance on benefits, housing and employment.	
Strengths of this approach	Limitations of this approach
● Many voices working together add strength and weight to a campaign ● Elected spokespeople are responsible to, and scrutinised by, the membership. They can be removed from post if the membership so wish	● Reaching consensus between diverse members is difficult ● Communication between members is time consuming and effortful: multiple methods are used and equal weight must be given to all voices regardless of their method of engagement

**Box 4.** Infrastructure – how to support and encourage autistic academics and activists (participatoryautismresearch.wordpress.com/).

The Participatory Autism Research Collective (PARC) was set up to bring autistic people, including scholars and activists, together with researchers and practitioners who work with autistic people. Their aim is to build a community network, where those who wish to see more significant involvement of autistic people in autism research can share knowledge and expertise.	
Strengths of this approach	Limitations of this approach
● While it is autistic-led, the group is inclusive of autistic and non-autistic academics ● The group specifically aims to support early career researchers and practitioners	● Autistic-led groups like PARC, operating independently of a host institution, may struggle to secure funding ● The membership is widely distributed across the United Kingdom, making collaboration and collective action difficult at times

**Box 5.** Empathy – how to build effective working partnerships (www.artscatalyst.org/jon-adams-konfirm).

*Konfirmation Systemisation: Rethinking Autistic Thinking* was an artist residency within the ARC Cambridge in collaboration with The Arts Catalyst, London and artist Jon Adams. Supported by Wellcome Trust funding, this residency led to poetry, image and a series of musical compositions made from fMRI machine noise.	
Strengths of this approach	Limitations of this approach
● Independent funding expands the range of opportunities for people involved ● The research group were influenced by the autistic artist’s presence, leading to a reconsideration of their views of autism	● To be successful, this requires investment of time on both sides and a willingness to challenge existing ‘knowledge’ or assumptions ● Independent funding may be hard to secure

### Topic 1: respect

One clear and consistent message from the autistic community and their allies was the need for autistic voices (incorporating all types of communication) to be heard and taken seriously at all stages of the research process. Seminar delegates reported that the lived experiences of autistic people – their ‘experiential expertise’ (Collins and Evans, 2002) – is rarely apparent in the context of autism research, though notable exceptions were identified (see Box 1). Perhaps, related to this, non-autistic academics at the seminars often had similar concerns about whether their expertise and perspectives would be respected by autistic delegates, especially those from outside academia. Indeed, a crucial component of engagement is to ensure that community representatives understand the context in which research (and indeed service delivery) takes place. Setting expectations about the limitations and timelines of research is essential to allow both partners work towards a shared goal. During the series, through dialogue, listening to one another’s viewpoints, recognising differences and accepting that there was not always agreement, mutual respect between autistic and non-autistic members grew from meeting to meeting.

How was this achieved? During the series, members of the autistic and autism communities played prominent roles in every event, including as co-applicants for funding, co-convenors, speakers, panellists and discussion group leaders. Community representation was visible, and in sufficiently high numbers (from about one-third to half of all in attendance) to give confidence to delegates from these groups. Moreover, substantial energy was put in to making each seminar as autism-enabling as possible by creating a suitable sensory environment and providing a quiet space. We reduced power inequalities between autistic and non-autistic contributors by including clear terms of reference for participation in the seminar programme, so that all delegates had a shared expectation of what the seminar would involve.^2^ In all seminar series materials, presentation titles and so on, language was selected which characterised autism in neutral terms – for example, we neither refer to autistic people as patients nor to autism as a disease or misfortune.

In this way, respect was made overt, allowing seminar delegates to move beyond traditional barriers and instead focus on both a need and an opportunity for working together to deliver benefits to autistic people and their allies. The result was that the series itself had become an example of participatory practice and the foundation for a community of informed, mutually-engaged and respectful stakeholders (within and beyond academia) building interactional expertise for autism research (Collins and Evans, 2002; Milton, 2014; Pellicano and Stears, 2011). This experience gave rise to three core principles of participatory research, which have formed the basis of a starter pack for researchers (Pellicano et al., 2017).

### Topic 2: authenticity

The seminars attracted many people who started from the point of view that participatory research is both morally right and practically beneficial. For this reason, much of the discussion quickly moved from a focus on basic barriers to participation (e.g. failure to provide an accessible environment) to more complex dimensions of high-quality engagement. The first key issue identified by delegates was tokenistic involvement, in which people’s input does not influence the outcomes. Engagement such as this, which serves only the purpose of ‘ticking a box’ – for example to meet a funder or ethics review board requirement – is not authentic engagement. At best, such approaches may fail to deliver useful results; at worst, they are insulting and excluding to autistic people and their allies, damaging the relationship between autistic people and researchers, and leading to non-participation in future research. It was agreed that to avoid tokenism, researchers should collaborate with community representatives who have expertise and experiences relevant to the specific topic under discussion; engage in open dialogue; listen and be prepared to learn from this expertise, make changes in response to feedback; and acknowledge the imbalance of power in most research scenarios.

Addressing an unequal power balance was the second key issue in quality engagement. Sometimes researchers using quantitative methods incorporate qualitative components into their project (to seek/reflect the views of autistic people) and assume that this constitutes good engagement. Yet, when conducting interviews or focus groups, the researcher still has almost total control – selecting participants, scripting questions, hosting the meeting, pooling data and drawing conclusions. Participatory working, including engagement prior to designing a study or seeking funding (see Box 2), is distinct from qualitative methods to answer a research question, in that it provides an opportunity for community members to shape the focus of the research itself. Continued consultation as research progresses can have a similar impact on research interpretation, dissemination and implementation.

A power imbalance may still apply when autistic and non-autistic academics work together: a clear message from the autistic academics at the seminar was about the disadvantages they face working in a high-expectation environment that often fails to recognise needs and provide suitable support (see *Infrastructure* below). To address power imbalances, people agreed that non-autistic researchers should consider meeting autistic people in places of their choosing, and opening up a dialogue that is not constrained by specific research questions. By spending time with autistic people, without an agenda or specific idea of what the researcher wants to do, we can build research questions on autistic input from the very outset. This argument aligns closely with the ‘slow science’ movement (Alleva, 2006), which emphasises investment of time and resources in the thoughtful consideration and selection of ideas before data collection. Even without such investment, attending autistic-led events such as *Autscape* in the United Kingdom or *Autreat* in the United States and reading the work of autistic bloggers are both ways to engage without imposing a priori assumptions and with only modest resources required.

### Topic 3: assumptions

The seminars addressed assumptions about autism that need to be challenged. Diagnostic criteria for autism include descriptions about ‘deficits’ of social-emotional reciprocity, non-verbal communication and relationships. Understanding of autism has largely moved on from attempts to characterise autism in terms of a single, universal ‘deficit’ and now recognises the diverse pattern of features. Nevertheless, in the context of community engagement, a belief that autism is characterised by – for example – an inability to understand others has been used as an excuse not to engage with autistic people (Pellicano et al., 2014a). Autistic delegates at the seminars reported that often an autistic person may not be considered a legitimate spokesperson for their wider community, even when acting as an elected representative of a community group. This phenomenon is probably exacerbated by the wide variety of autistic dispositions: there is concern that verbally and cognitively able autistic adults cannot speak on behalf of those who have intellectual disabilities and/or significant barriers to communication.

In contrast, our experience demonstrates the opposite. The seminars heard from multiple examples of autistic people supporting their peers, such as autistic advocates aiding members of their community to access health and social care (see Box 3). That said, some autistic people may (understandably) object to being expected to advocate on behalf of ‘their community’ – we should not mistake a drive towards a participatory research agenda for a pressure on individual autistic people to become advocates and activists. In addition, during the seminars, aspects such as recognition of intersectionality (the overlapping disadvantageous influence of multiple characteristics subject to discrimination – such as race, sexuality and neurodiversity^3^), and consideration of the needs of other neurodivergent people (e.g. people with attention-deficit hyperactivity disorder (ADHD), depression, dyspraxia, epilepsy or non-speaking autistic people) was consistently flagged by autistic delegates when sometimes overlooked by the non-autistic people present.

### Topic 4: infrastructure

One of the key requirements for effective participatory research is supportive infrastructure. Delegates from within and outside academia at the seminars were united in their perception that the basic infrastructure of research – especially scientific research – is not conducive to participatory working. For example, some academics, working from a traditional notion of objectivity, were concerned that the scientific endeavour could be biased by engagement with autistic partners. To those, we offer that serious biases – for example, towards maintenance of the status quo – can occur when research takes place without community influence. One example might be the interpretation of both increased and decreased activation of brain regions in an functional magnetic resonance imaging (fMRI) study as reflecting autistic ‘deficits’ (Dawson and Mottron, 2011). Critical reflection on the meaning of experimental tasks used in research, and involvement of autistic people in interpretation of data, can help to avoid the automatic attribution of deficits to data that are, in and of themselves, value-neutral.

One manifestation of this culture is that funder priorities do not normally include community participation, or if they do, this is rarely more than tokenistic. Funding strategies are highly influential on the direction of research and the methods used. Engaged funders could help to effect culture change by requiring evidence of relevant community consultation on all submitted proposals, incorporating lay reviewers into their evaluation process, and following up on researcher ‘Impact’ statements to check that proposed dissemination and implementation plans have been delivered. These measures require academic and non-academic reviewers to be sufficiently skilled to evaluate the quality of proposed participatory activities. Without funder endorsement, individual researchers attempting to build in high-quality engagement may be demotivated to do so. It is true that quality engagement takes time and costs money, which may make proposals less competitive if the engagement component is not valued by the funding body. Researchers may be able to influence funder attitudes by persistently incorporating participatory methods into their proposals and by requiring these when asked to review proposals.

Where engagement is supported by funders, researchers need to ensure that they cost consultancy fees for individuals and/or contributions to autistic-led organisations into their proposals. Suitable payment, recognising the professional and personal expertise required for the role, and the associated level of responsibility in relation to project aims, is a key way to demonstrate respect and address the power imbalance. However, we also note that even when the funding is available the administrative logistics of making a payment to a ‘lay consultant’ can be very challenging. Involve – a UK organisation for ‘patient and public involvement’ in health research – has published useful guidelines^4^ though in some cases, academics may find these conflict with their grant reporting requirements or University procedures.

Autistic researchers are significantly disadvantaged by institutional and wider research infrastructure, which has historically failed to recognise neurodiversity and often serves to promote research by already privileged groups. Current attempts to improve equality and diversity in the United Kingdom higher education sector (e.g. *Equality Challenge Unit*^5^) should be extended to incorporate the issues faced by neurodivergent academics. Some universities are already making steps in this regard, since disability is a protected characteristic under law in the United Kingdom and many other countries ^6^. Nevertheless, partners in the seminar series described ‘institutional ableism’ built into university systems and difficulty finding appropriate post-graduate supervision that recognised their needs in relation to the work (Martin, 2010). Best practice in this area has often been led by the neurodivergent community, as in the founding of the open-access journal *Autonomy* (Arnold, 2012). Despite these strides, a change to academic infrastructure is a necessary, but not sufficient, step if we wish to achieve higher rates of autistic leadership of research projects relating to autism.

### Topic 5: empathy

The double empathy problem (Milton, 2012) highlights the issue of ‘mutual incomprehension’ that exists between some autistic and non-autistic people, in all walks of life. Indeed, there is a growing body of evidence which demonstrates empirically that non-autistic people may fail to comprehend autistic people (Sheppard et al., 2016), or negatively judge them based on minimal evidence (Sasson et al., 2017). If not addressed, this lack of shared understanding presents a significant barrier to effective research collaboration. Thus, even those researchers who feel motivated to engage with the autistic community may find themselves unsure about where, or how, to start. In particular, autism researchers may be fearful that autistic people will say something they disagree with or ask them to do something in a project that they cannot easily do. The irony of this should be obvious: researchers have been asking autistic people to put up with both of these for decades.

Nevertheless, it is true that sometimes autistic people will be very frank in their judgements about research plans and processes, and fail to conform to social norms. This can be challenging for non-autistic researchers, but should also be viewed as an opportunity. Open dialogue about the focus and methods of research, with autistic people and their allies who are not researchers, can not only help to contextualise the work but also educate communities about the realities of the research process. This is true knowledge exchange, in which both parties gain new insights from the interaction. While consensus will not always be achieved, the process of dialogue and engagement remains valuable as a source of mutual learning. Building up trusting professional relationships between researchers and community members makes this learning more direct and easier for both parties. Over the course of the seminar series, we have been able to facilitate such relationships, leading to the beginnings of a new, engaged ‘community of practice’ in autism research in the United Kingdom (Hart et al., 2013). Crucially, such dialogue will not necessarily result in consensus, but mutual learning is a process rather than an outcome.

### Next steps for participatory autism research

This report of themes emerging from the *Shaping Autism Research* seminar series aimed to focus on solutions rather than barriers to participatory research. Nonetheless, there is still much work to be done. We characterise this work as falling into two categories: *supportive environments* and *methodological challenges.* The first category describes various activities already touched on above, which are necessary to build a culture where autistic people and their allies can take on active, meaningful roles in research. These include: changing the language we use to describe autism (Gernsbacher, 2017; Kenny et al., 2016); modifying or identifying physical spaces to enable autistic participation; and adapting the structures and bureaucracy of academia to facilitate autistic involvement and leadership in research. In so doing, we should draw on the experiences of pioneers within (Frauenberger et al., 2013; Gillespie-Lynch et al., 2017; Mason et al., submitted; McConachie et al., 2018; Nicolaidis et al., 2013; Parsons et al., 2013) and outside (Brett et al., 2014; Rose, 2003) autism research. Showcasing these examples may help to draw in community representatives who are sceptical about the capacity for improvement in the research establishment. Another way to create a supportive environment is to improve the research literacy of the community by sharing insights into the research process and enabling access to the scientific literature.

*Supportive environments* can also be applied to making a space in which to welcome those academics who may feel that the participatory research agenda does not recognise the constraints and priorities of their research. Some – such as those engaged in basic biological research – may feel that their laboratory-based projects are far removed from community concerns and thus that engagement is not required. Early career researchers may identify with the agenda, but lack the skills, resources or support to develop this aspect of their work. Expanding our scientific ideologies to make room for participation is a challenge, but we must reach beyond the small, but growing, network of autism researchers who do incorporate engagement as a matter of course in their research.

*Methodological challenges* encompass those issues which are not addressed by current roadmaps for participatory research. A prominent example is the question of how to capture the voices of autistic people who are not easily integrated into even the fledgling participatory research structures available at this time. This includes autistic children, those with intellectual disabilities and those who do not speak. While innovative practices are being developed (Gaudion et al., 2014; McDonald and Stack, 2016; Ridout, 2014 see also Pellicano, 2018), we remain far from achieving meaningful, let alone routine, integration of these voices into research.

Another difficulty, not restricted to the autism field, is how to balance individual and collective opinion, including how to respond to disagreements within and between groups (Fletcher-Watson et al., 2017a; Milton, 2016). Historically, parents of autistic children have been listened to somewhat (Silverman and Brosco, 2007), and autistic people less so. Parents, like practitioners and third-sector workers, can advocate on behalf of their children and may often be stakeholders in research themselves – they should be included in the participatory agenda (Fletcher-Watson et al., 2017b). For this reason, we have referred throughout to engagement with both the autistic and the broader autism community. Nevertheless, consultation with parents of children on the autism spectrum should not happen to the exclusion of autistic people themselves. Moreover, when consulting with, for example, both autistic adults and parents regarding a study with pre-schoolers, how should researchers handle any conflicting advice from these groups?

Even within a stakeholder category – for example, among autistic people – there will be a broad diversity of views. A particular challenge may be the case when an individual from within the autistic community is advocating for a position which reflects their own view, but is not well supported by a broader constituency of autistic people. That said, it is misleading to suggest that consultation with members of the autistic community gives non-autistic researchers access to a consistent ‘community view’. One way to address this is to ensure that any focused consultation with a specific individual is complemented by wider engagement – perhaps via social media or at events (while recognising the bias that can arise from these engagement methods too). Ultimately, despite the challenges described here, it is hoped that the growing autistic rights movement and increasing prevalence of participatory research will enable people to recognise and respect differences rather than attempting to force a consensus (Milton et al., 2012).

In addressing methodological challenges, and building supportive environments, we encourage researchers and others with relative influence and power (e.g. senior practitioners, policy-makers and funders) to work with autistic-led organisations in the United Kingdom, such as the *Participatory Autism Research Collective, All Wales People First* and *Autism Rights Group Highland* (Chown et al., 2017). Such groups may have elected representatives who can reliably speak for a larger community. Moreover, by raising their profile, we can provide a focus for autistic individuals who wish to be heard. In fact, it is worth noting the leadership role which has been played by autistic advocates and activists in pioneering the neurodiversity movement. The number of autistic-led organisations, publications (e.g. Autonomy; Beardon, 2017; Lawson et al., 2017; Murray, 2005), online communities (e.g. wrongplanet.net) and events (e.g. Autistic Pride, Autreat and Autscape) is a testament to the energy and dedication of this community. Such initiatives provide opportunities for researchers to make connections which may yield significant benefit to all involved.

### Limitations

The report presented here should be viewed as a way to open up further discussion about the role, and delivery, of participatory methods in autism research. One limitation is that this discussion focused often on social sciences and psychological methods, rather than on biological and neurological research. There may be specific barriers that apply in this content, not discussed here, such as the technical knowledge required to engage in a productive partnership with members of the autistic community. In the medical research field, the work of groups such as Involve^7^ could provide a model to follow, though the mapping between engagement with patients and research with autistic people may be inadequate.

We do not present a series of empirically-derived recommendations but instead report on the intensive considerations of a small but diverse group, drawing on the broader discussions across an entire seminar series. The seminar series was not fully inclusive to people with a learning disability, and no non-speaking autistic people took part. These key demographics were not represented, though parents and other allies of such individuals did take part – including in co-authorship of this publication. While there is guidance on how to start out in participatory research (Pellicano et al., 2017), materials to enable this burgeoning community of practice to extend and improve their work, and specifically to include a wider diversity of autistic perspectives, remain lacking (though see Scott-Barrett et al., 2018).

## Conclusion

While our seminar series was created around a series of research areas, the topics which emerged from the six events concern the *why* and *how*, more than the *what* of research. Differing perspectives from the autism community and research community were expressed, enabling institutional assumptions to be challenged, and ultimately articulating a common vision for mutual and equal engagement. Our collective hope is that the foundations laid throughout the *Shaping Autism Research* seminar series will lead to a greater, co-created knowledge base for the better integration of community perspectives in research. This will not come easily and can only happen with considerable effort from relevant communities and stakeholders, as well as evaluation of the effectiveness of participatory methods. The opportunity is to create a burgeoning, merged community of research practice, including autistic and non-autistic people and other partners who work collaboratively to create facilitative environments and resolve important, relevant questions. The research evidence developed in this context should then be implemented, providing structures to support autistic people and their allies, and is more likely to achieve this goal having been co-created. Meaningful participation in autism research can help us make a better future for autistic people, together.

## Supplemental Material

AUT786721_Lay_Abstract – Supplemental material for Making the future together: Shaping autism research through meaningful participation Click here for additional data file. Supplemental material, AUT786721_Lay_Abstract for Making the future together: Shaping autism research through meaningful participation by Sue Fletcher-Watson, Jon Adams, Kabie Brook, Tony Charman, Laura Crane, James Cusack, Susan Leekam, Damian Milton, Jeremy R Parr and Elizabeth Pellicano in Autism

## Notes

1. Identity-first language (e.g. autistic people) is the preferred language of many people on the autism spectrum (see Sinclair, 2013) and their parents (Kenny et al., 2016). It is also the preferred language of the autistic co-authors of this report. We, therefore, use identity-first terminology in this article.

2. A template programme for autism-enabling events is available on www.shapingautismresearch.co.uk.

3. Neurodiversity is defined as the range of differences in individual brain function and behavioural traits, regarded as part of normal variation in the human population, encompassing diagnostic categories such as autism.

4.
http://www.invo.org.uk/posttypepublication/involve-policy-on-payments-and-expenses-for-members-of-the-public-including-involve-group-members-february-2016/

5.
https://www.ecu.ac.uk/

6.
http://www.ncl.ac.uk/diversity/assets/documents/equality_strategy_2014-16-jh.pdf

7.
http://www.invo.org.uk/
