# Supplementary material for: Making the future together: Shaping autism research through meaningful participation
Source: Autism. 2018 Aug 10;23(4):943–53. doi: 10.1177/1362361318786721 (PMC6512245; doi:10.1177/1362361318786721)
Supplement: AUT786721_Lay_Abstract – Supplemental material for Making the future together: Shaping autism research through meaningful participation [file AUT786721_Lay_Abstract.pdf]

Lay Abstract

autism

DOI: 10.1177/1362361318786721

Making the future together: Shaping autism research through meaningful participation

Sue Fletcher-Watson, Jon Adams, Kabie Brook, Tony Charman, Laura Crane, James Cusack, Susan Leekam, Damian Milton, Jeremy R Parr and Elizabeth Pellicano

"Participatory research" refers to research in which members of a community work with academics, to achieve shared goals. These methods can produce information that is relevant to people's lives and thus likely to have a positive impact. Although there is a large and growing body of autism research, it doesn't always influence the community effectively – for example, by changing how autistic children are taught in schools. In addition, a large survey study showed that autistic people and their families, as well as professionals who work with them, want research to focus more on things that affect their lives. In this context, we think participatory autism research can make a positive difference. In order to develop this idea, a UK seminar series was organised by autistic and non-autistic people. This paper reports on what we learnt during the series. We describe five topics that we think are important for participatory research in autism: Respect, Authenticity, Assumptions, Infrastructure and Empathy. We illustrate each topic with a specific example from within and beyond research, to inspire new practices. We call for more participatory autism research, including greater autistic leadership of research. Such work, if delivered to a high standard, is likely to lead to better translation into schools, clinics and the community, with benefits for autistic people and those who support them.
